# Process, Not Product: Investigating Recommendations for Improving Citizen Science “Success”

**DOI:** 10.1371/journal.pone.0064079

**Published:** 2013-05-15

**Authors:** Amy Freitag, Max J. Pfeffer

**Affiliations:** 1 Duke University, Marine Science and Conservation, Beaufort, North Carolina, United States of America; 2 Cornell University, Development Sociology, Ithaca, New York, United States of America; UCSD Medical School, United States of America

## Abstract

Citizen science programs are increasingly popular for a variety of reasons, from public education to new opportunities for data collection. The literature published in scientific journals resulting from these projects represents a particular perspective on the process. These articles often conclude with recommendations for increasing “success”. This study compared these recommendations to those elicited during interviews with program coordinators for programs within the United States. From this comparison, success cannot be unilaterally defined and therefore recommendations vary by perspective on success. Program coordinators tended to have more locally-tailored recommendations specific to particular aspects of their program mission.

## Introduction

Science is a process that cannot be completely understood without fully understanding the actors, their interactions with each other and nature, and their context [1]. Citizen science is a means of engaging the public in the scientific process and serves as a means to recognize the wider contexts of science, including culture and policy. As a result, citizen science harnesses society’s recognition of science as a process in order to provide additional benefits outside of pure scientific results [2]. Citizen science programs are evaluated in a number of forums, including in the discussion sections of academic literature and during personal reflections between coordinators. Both literature and coordinator reflections document many types of citizen science benefits, from scientific and educational perspectives [3].

The recent upswing in both quantity and scale of citizen science projects can be attributed to an increase in enabling technologies, the need for large-scale datasets, and the push for outreach by funding agencies [4]. The increasing number of programs reflects broad realization of the multiplicity of benefits citizen science offers. Social benefits include educating the public in science and scientific thinking, inspiring appreciation of nature, and promoting support for conservation initiatives [5]. Scientific benefits include coverage of large spatial scales, long time series, data from private land, and labor-intensive data that would otherwise be expensive to collect [6].

The science studies literature describes these benefits collectively as resulting from recognizing the value of non-scientific expertise (though caution they are not always achieved). Citizens experience the world in ways outside the traditional scientific method, allowing physical and temporal access to the question at hand that is necessary to provide these benefits and others. The perspectives citizens bring from their individual ways of knowing can offer creative approaches to scientific problem-solving and connect scientific information to policy applications [7]. Ways of knowing, often termed ‘local’, ‘indigenous’, ‘traditional’, ‘citizen’ knowledge, or schemata, offer varied framings of particular issues in line with a critical realist philosophy that multiple perspectives are needed to fully describe the world [8]. Incorporating diverse ways of knowing into the analysis of a given issue increases understanding of the issue and offers solutions better tailored to the full context [9], [10]. One of the main differences between citizens and scientists is the tendency for citizens to rely on procedural not substantive schemas (that is, they focus on how to learn instead of what is learned), hence citizens focus on process when legitimizing expertise [11].

The focus on process by citizen science is a small-scale example of a larger societal phenomenon recognizing many forms of expertise in science – termed “democratizing science” [12] or the “third wave of science” [13]. Though scholars describe details of the phenomenon somewhat differently, the hallmark is the breaking down of walls between scientific experts and the general public. Through this phenomenon, the scientific process expands to incorporate segments of the public existing outside ivory towers and government halls. The motives of the democratizing science movement directly respond to the “scientization” of policymaking, or legitimization by using science-based decisions. Expanding the base of what is considered scientific expertise therefore also expands political power [14]. This is particularly important in the environmental sciences, where many regulations mandate “best available science” (eg. Clean Water Act, Clean Air Act) and democratizing science authorizes more types of information to be used for political decision-making.

The benefits of citizen science and democratizing science originate from emphasizing process – specifically by spreading power derived from expertise among more people, creating a scientifically educated public for future scientific development, and connecting science to everyday life. Citizen scientific co-production of knowledge parallels benefits from co-production of policy, or co-management [15]. Both forms of co-production focus on process as a performative practice, normatively creating new types of citizens. In the case of citizen science, these citizens sit along the boundary between science and the public [16] and perform as better ecological citizens than either average citizens or scientists [3]. Therefore, knowledge co-production may serve a similar function as co-management in creating distributive justice and legitimate outcomes (as perceived by the public).

Theorizing knowledge co-production and nesting citizen science within the broader movement to democratize science are recent phenomena. Many citizen science programs pre-date scholarship on the matter, hence there are concerns when attempting to achieve both scientific and social benefits in one program [17]. For instance, the traditional scientific method focuses on data quality and advancing scientific theory; both of these become less important in citizen science efforts. The metrics of success are not as unilaterally defined in citizen science [18].

In a fully democratized science, ‘success’ depends not only on the quality of the product but also the process. There is still a demand for the “best available science”, interpreted as high-quality data and building blocks for scientific theory development, especially from the established scientific community [19]. Citizen science does not replace this definition of “best available science” but adds a new dimension. The broader definition includes wider participation, broader impacts to society, and chances for many perspectives to add their voices to the final analysis. The push for procedural success comes from the citizen scientists as well as the program coordinators, who likely attend to a multiplicity of program goals [3].

### Hypothesis

Since citizen science efforts predate the movement for a democratized science and therefore may not employ the broadened definition of success, academic literature might leave out citizen recommendations for improving that success. Specifically, published articles favor the hypothesis-testing model of science that many citizen science projects do not easily fit into, suggesting the need for a more thorough review [4]. Through a literature review and interviews of project coordinators, we investigated whether recommendations in the literature accurately inform citizen science projects in the field. We also investigate perceived success by asking whether the project’s data (the product) meets the goals of the program (the process).

## Methods

### Phone Interviews

We identified leaders of citizen science projects through a Google search using the search terms ‘volunteer’ and ‘monitoring’, followed by systematic random sampling of the search results beginning on a random page, using programs that identified as citizen science within the United States on their webpage. The search was intentionally structured this way to focus on long-term, environment-based citizen science programs. This left out other disciplines’ citizen science projects (like computer-based games for biochemical structures or astronomy identification); of course, we ran the risk of missing programs that did not identify themselves using this particular terminology, but we achieved an adequately sized group of programs for analysis. We contacted the phone number listed for either the program coordinator or primary investigator, with an overall 50% response rate for a total of 19. The non-responses were all from incorrect contact information: disconnected phone lines while not in field season (as determined post-analysis) or people no longer affiliated with the program. The responses may therefore favor programs with year-round staff and larger programs with up-to-date websites.

Structured interviews lasted around 20 minutes each following an interview guide with questions addressing program mission, daily function, and recommendations for other programs to be successful. We coded answers according to code trees directly addressing our research questions, adding sub-codes to each tree as they emerged. We also took notes during the interviews, especially of tangential or explanatory information, for later analysis coded using grounded theory [20] in order to incorporate unexpected themes not directly addressed in our questions. A formal written exemption from the need for IRB Review was received from the Cornell University Institutional Review Board, following exemption guidelines of a) all adult respondents, and b) no identification information permanently recorded. The IRB approved the protocol including oral consent from respondents since communication was via phone only.

### Literature Review

We identified articles for review through a search in three databases covering education (ERIC), environmental science (Web of Science), and sociology (Sociological Abstracts). Search terms included ‘citizen science’, ‘volunteer’, ‘assessment’, and ‘monitoring’. We reviewed each study that occurred within the United States and coded them according to the following questions (derived from the interview script for ease of comparison); articles answering none were dropped from review, leaving a total of 67:

- Did the author consider the study successful as determined by the stated purpose of the study?
- Did the author consider the data accurate? At what level?
- What are the recommendations of the authors for other investigators hoping to use citizen science?

## Results

### Top Recommendations for Success

Program coordinator interviews reached programs that were on average 12 years old, with variation from one to 38 years. Programs size varied, having an average of 279 volunteers ranging from 15 to approximately 5000 (see table 1 for attributes). About half of the programs (53%) had volunteer coordinators in addition to overall program coordinators responsible for volunteer training, recruitment, supply distribution, and other details. About half (58%) also had established collaboration with local experts such as university professors or government agency scientists. Volunteer training was often mandatory (63%), longer than 3 hours (42%), and contained a handbook for reference (68%). Most programs (68%) also reported consistent commitment from their volunteers, with people participating year after year once recruited. All but one program made an effort to make their data publicly available (the one that did not was attempting to protect locations of the endangered species they monitor) through newsletters and internet databases. Most of these databases were newly constructed and many were still “under improvement”. More than half (58%) of the programs published in the scientific literature, often through their scientific collaborators; these were mostly from coastal programs. Note that here and in the following analyses, percentages represent approximations because of the small sample size. One coordinator represents 5.3% of the total number of respondents, so for generalizing to nationwide trends, presented percentages could reasonably be +/−5%.

**Table 1:** Attributes of volunteer programs.

	Number	Percent
Average age of program	12	n/a
Volunteer coordinator	10	53%
Professional collaboration	11	58%
Mandatory training	12	63%
Consistent volunteer commitment	12	63%
Data publicly available	18	95%
Published in scientific literature	11	58%

Total n = 19.

The top recommendations from program coordinators, mentioned by at least a quarter of respondents, are as follows: 1) collaborate with experts, 2) have a consistent methodology, and 3) present data to policymakers (see table 2). Answers about recommendations were accompanied by explanations as to why those topics were important and specific examples of how these recommendations would have helped their program in the early years; these were more important than the short version, so further discussion will focus on these explanations. These details primarily exposed the utility of specific recommendations. For instance, collaboration with experts included access to expert advising overall, efficient use of small state grants, technique sharing, and generating new and applicable research questions. Respondents also directly linked collaboration to the #2 recommendation, consistent methodology, crediting the collaboration with the means to create consistent methodology both within the program and within the larger network of people studying the same ecosystem.

**Table 2:** Summary of the top recommendations from both the phone surveys and literature.

Surveys	Percent	Literature	Percent
Collaborate with experts	53%	Collaborate with experts	48%
Have a consistent methodology	47%	Present data to policy influencers	45%
Present data to policymakers	39%	Have a consistent methodology	40%
		Have a standardized training program	39%

Over half (57%) of the literature used in the meta-analysis reported results from citizen science programs existing for more than five years. Most of the funding for these programs came from either government (30%) or academia (43%), which is often funded through grants from the National Science Foundation. The stated purpose of these articles, with the exception of two, was to aid the development of a citizen science program in a particular location or habitat. These demographic traits mean that most of the articles discussed programs that had long-term history and long-term goals.

The top recommendations for the literature are as follows: 1) collaborate with experts, 2) present data to policy influencers, 3) have a consistent methodology, and 4) have a standardized training program (see table 2). Each of these recommendations was mentioned by about half of the articles and was clearly more prevalent than the remaining topics, which had at most a quarter of the studies mentioning them. Some of these remaining topics have emerged in previous reviews: need for data verification, field checks, and large data sets [21]. Unlike the phone interviews, many of the articles did not elaborate on their recommendations, so the motivation and utility of these efforts for a specific project are not made clear.

### Differences between Literature Review and Surveys

Although the top recommendations were shared in the literature and surveys, there are a few notable differences. First, the fourth recommendation from the literature – to have a standardized training program – was only mentioned by one survey respondent, who commented that the only reason training was an issue for his program is because they hadn’t instituted standardized training from the inception of the program. Several forest monitoring programs mentioned the need to “get it right the first time”, incorporating the idea that initially setting up the program correctly, on a number of facets, is more important than any one aspect of program function.

The survey respondents created a much longer, more varied list of suggestions than the literature review generated, generally tailored to specific challenges their program had faced. Though these results were initially coded to the same categories as those found in the literature review, this specificity is notable because they are written for new program directors to be successful in similar programs. The literature review recommendations were aimed at a more general audience. For example, the top recommendation of the need to collaborate with experts was to “look at other existing programs”, for “networking, the way to answer specific questions”, or as “crucial to pull resources together effectively”. Many groups also stated they “absolutely would not be able to do anything [without partnerships]” and that meeting the other recommendations followed as a consequence of collaboration. These specifics better describe the nature of the collaboration, which experts are involved, and what products are expected as a result.

The recommendation of needing consistent methodology was often phrased in the context of a particular challenge the primary investigator faced. For example, some doctorate-holding volunteers in a Gulf of Maine monitoring program felt the need to revise existing protocols on the fly; the program coordinator attributed achieving consistent methodology in this case to enforcement and emphasis on existing written protocols. In contrast, the methodology challenge to most stream monitoring programs is the level of taxonomic detail required to follow the protocol; they instituted field handbooks in response. In yet other cases, practice made perfect, where more than 3 hours of training were required before volunteers could measure and officially record data.

The final recommendation from the literature, connecting to policymakers, was more straightforward. Still, there were some groups that approached the citizen science process as objective scientists and felt the data should speak for themselves. In these cases, they wanted to be able to hand over raw data to state or local agencies to include in larger databases used for decision-making. In other cases, the approach was through advocacy, where digested results would be presented at public meetings, annual volunteer meetings with invited policymakers, or included in letters to elected representatives.

### Data Enough for Success?

Both sources of recommendations direct future programs toward behaviors for success. However, not all programs had the same definition of success. The mission of each program was not purely about creating scientific information, but about some combination of education, restoration, stewardship, and community-building. As with the recommendations for success, the surveys revealed more nuanced answers about mission or purpose. Many respondents hesitated a moment before classifying the success of their program, given the mission statement.

“Success” also depended on the larger socio-ecological context. One program coordinator stated that it is “hard to measure success” when watching floundering lobster nurseries despite their program’s efforts. She knew that they had saved at least six nurseries from development projects, but still had a hard time classifying the last few years of her program as ‘successful’. Others respond to context by changing their mission. A coordinator doubted the relevance of their mission, expressing uncertainty as to “who we should become next… we’re at a crossroads”. This program could not have success before defining their new mission. One grassland program described the work of the group as on an “as we can handle basis”, shifting from one mission to the next as need arises.

Stated program purposes fell into 6 general categories: mapping species distribution, increased stewardship, restoration, baseline data, tracking trends, and “not sure”. Examples of these missions include “create a habitat for the future”, take “just a snapshot”, “actively changing the program based on what the public needs”, and “to get data, but awareness and stewardship”. 84% of coordinators classified their program as having met its purpose with slightly less (68%) stating confidence in their data (or, in many cases, at least some of their data).

Because there are a multitude of purposes, the questions “do you consider yourself successful” and “is your data reliable” do not necessarily address the same thing. Though most coordinators were confident in their data, that confidence came with the caveat that the data may not be professional, but results are certain “from the sheer amount of time out there”. Others recognize that not all their data is reliable but they are “along the right steps” and have some data to share. The extreme ends of the spectrum also existed, creating data that “absolutely and exceeded” certainty or “data, maybe…education, certainly”.

The articles reviewed claimed success most of the time (97%), with their overall goal being scientific in nature. Part of this result may be due to the tendency for scientists to only publish positive results, leaving negative results to the grey literature or a filing cabinet [22]. Some of the articles presented the use of citizen science as a method – for example, “A simple method of measuring beach profiles” [23] - not emphasizing the other benefits that may accompany or dominate a citizen science program. Still others recognized problems in using purely citizen-derived data, such as “Monitoring the distribution of pond-breeding amphibians when species are detected imperfectly” [24], but still considered those studies a success. In the words of one forest study, “since the program’s purpose is to track major changes to the forest structure as measured by dominant canopy trees, it may be irrelevant whether or not volunteers can distinguish *U. americana* from *U. rubra*” [25]. The data collected from these programs may not be at the level of a trained scientist, but they appear to be good enough, especially for the program’s purpose.

### Building Community

The concept of community, both inside the program and within the program’s region, was mentioned by many of the respondents in different contexts. When asked about program demographics, many coordinators said a better question to ask is how many people from the community are involved. Especially for programs in small towns, coordinators were proud of the fact that nearly every resident participated in some way in the program. They stated that the community-building process strengthened their program overall. One program held an annual summit, which the coordinator described as putting “context to their work” by presenting efforts to the community and showing volunteers tangible results.

## Discussion

At first glance, literature and interviews yielded similar recommendations – but only in the general sense. Interviews revealed a much more varied list of suggestions leading to less clear “top” suggestions (which were reported here for comparison). The recommendations out of the literature employed blanket statements strategizing scientific successes. Such recommendations need to be filtered through the particular context of a program, be that mission priorities, habitat, volunteer demographics, or something else. The more specific suggestions made by program coordinators emphasized particular parts of the process – collaboration for resources or new research questions rather than legitimacy through ties to established experts or data-checking. Thus, recommendations from the literature may be helpful when planning the broad aspects of a program but not as helpful in the first few struggling years that every program goes through.

Similarity in these broad-scale recommendations is logical because successful process often leads to successful product – in this case, reliable data. The difference between the recommendations from the literature and the surveys lies in recognizing the mechanism of achieving that end and the concomitant benefits. For instance, presenting data to policymakers may at first seem tangential to the scientific goal of a program, but through raising excitement in the program, such action ensures more long-term volunteers that maintain the strength of the program and the integrity of the data [25].

The main difference in the broad-scale recommendations is that the standardized training emphasized in the literature did not emerge as a priority among program coordinators. Standardized training may already be expected in the structure of a volunteer program since multiple people are collecting data to later be compared. Perhaps this is so obvious an expectation that it need not be said in formalized recommendations. Alternatively, the term ‘standardized training’ might imply that volunteers are merely “data monkeys” with little input in their own educational experience. Since volunteers may expect constant interaction with scientists and other volunteers as part of their learning experience – and a common mission of the programs – standardized training may serve only as a minimal educational baseline; therefore it is prioritized in the literature recommendations focused on data, but not by program coordinators who aim for increasing education.

The programmatic mission as determined through the phone surveys varied, making the definition of success different for each program. Only one of the intended program purposes (collecting baseline data) was data-focused, defining success by the quality of data collected. Others were process-focused, such as tracking changes over time or increasing stewardship in participants. The process was more often successful than the product (data) and some of the respondents pointed out that the data is “good enough” or not the main focus of the program. This is in stark contrast with the published studies, many of which discuss citizen science as a method, evaluated against traditional methods by the same metrics of success – data quality.

Even though most of the programs were confident in the reliability of their data, most had also dismissed at least some of the collected data as unreliable. Program coordinators reported having many still-unpublished results, either because they were negative or not reliable enough. Consequently, the publications resulting from these types of programs represent a self-selected group with enough positive results to write about. The pool of literature found for the review likely represents a similar fraction of programs in existence. These programs had some component of their mission focused on creating baseline data. The recommendations from these programs found in the literature therefore refer to specifics that address only one type of mission, which is not representative of the myriad programs practicing.

### Conclusion

Success in citizen science programs is defined by the particular mission statement guiding the program, which is more likely to focus on the scientific process than purely on the results. Of course, a strong and successful process is more likely to lead to reliable data, but not necessarily in all cases. The differences in the specificity of recommendations observed between the literature and surveys can be explained by what they were making recommendations for. The literature recommendations represent a subset of projects with successful missions relating to providing data while survey recommendations were more sensitive to other aspects of missions and incorporated more struggling programs. Thus the focus on process should be made explicit when making recommendations, advertising or fundraising for citizen science groups.
